# Pitfalls in body fluid identification – age independent DNA methylation markers for vaginal secretions and menstrual blood in sexual assaults

**DOI:** 10.1007/s00414-026-03717-0

**Published:** 2026-02-19

**Authors:** Helen Konrad, Leandra Jürgens, Paula Hiekel, Sophie Fertyk, Daniela Wübbe, Benno Hartung, Micaela Poetsch

**Affiliations:** 1 Institute of Legal Medicine, University Hospital Essen, Essen, Germany; 2 Medical specialist in gynecology and obstetrics Dr. med. C. Beckemeier, Dr. med. F. Broer-Boos & Dr. med. D. Wübbe, Oer-Erkenschwick, Germany; 3 Institute of Legal Medicine, University Hospital Essen, Hufelandstr. 55, Essen, D-45122 Germany

**Keywords:** DNA methylation, Body fluid identification, Stain identification, Body fluids, Vaginal secretion, Menopause, Crime scene investigation, Sexual assault, Hormone status

## Abstract

In recent years, determination of cellular origin of DNA has been acknowledged as an important tool in forensic genetics. It complements identification of the DNA donor by STR analysis, since it could provide further information about the origin of DNA found at a crime scene. A workflow (body fluid identification (BFI) workflow) developed in 2023 for determination of seven different body fluids in forensic genetic casework by DNA methylation analysis has been very helpful in context with sexual assaults, especially rapes. The verification of vaginal secretions and/or menstrual blood, for which no (or no reliable) preliminary tests or rapid tests are available, can be decisive in these cases. During case work, it was hypothesized that the methylation pattern of vaginal secretions cannot be assumed to be universal but rather might depend on the female’s hormonal status or age. Validation studies have confirmed this assumption. Both, vaginal secretions from young girls before sexual maturity and from women who have already reached menopause showed a deviation from the established and validated DNA methylation percentages of the BFI workflow published in 2023. For this reason, additional vaginal marker V1(cg09765089) was established and validated in this study, so that in combination with marker V2 already published Konrad et al., (Int J Legal Med 137(6):1683–1692, 1) 99% of vaginal secretion samples could be identified, regardless of woman’s age or hormone status. Additionally, the new blood marker B6 (cg17518965) was integrated in the eBFI workflow (extended body fluid identification workflow), as the previous blood marker could not provide unambiguous identification of the target secretion in children in the context of sexual assaults. Overall, we have analyzed 453 samples in this study using bisulfite conversion and pyrosequencing. In summary, our revised eBFI workflow is a simple and useful tool for forensic analysis to identify vaginal secretions and/or menstrual blood in the context of sexual assaults, regardless of female’s hormonal status or age.

**Supplementary Information:**

The online version contains supplementary material available at 10.1007/s00414-026-03717-0.

## Introduction

 The great interest in methods for body fluid identification in criminal investigations shows the potential and relevance of information obtained by analyses on the (cellular) origin of DNA. In forensic genetic reports, there is a hierarchy regarding the hypotheses to be evaluated. After answering the question about the individual from whom the DNA sample originates (sub-source level), the second highest priority is about the origin of the DNA trace (source level) [2]. The results can allow reconstruction of the course of events and verify statements of involved people [3, 4]. In addition to routine immunochromatographic rapid or preliminary tests for determination of blood, saliva, seminal fluid and urine [5, 6, 6, 7], various RNAs [8, 9] or specific methylation patterns are used as biological markers in current studies [1, 10, 11]. These also allow identification or differentiation of other secretions and tissue like vaginal secretion, menstrual blood, nasal secretion, nasal blood or skin [1, 8, 10, 11, 11, 12, 12, 13].

DNA and RNA analyses for body fluid identification are based on quantitative detection of specific RNAs or on determination of methylation percentage of specific CpG motifs. Both factors depend on the epigenetic regulation of the cell types present in secretions or tissues [14].

In our recent study [1], a workflow (BFI workflow) was developed to identify or differentiate between a total of seven different body fluids based on methylation percentage. Since then, this method has proven its worth in our laboratory not only for differentiating between various bleeding scenarios (e.g. nosebleeds vs. blood), but especially in context of sexual assaults by identifying vaginal secretions or menstrual blood. The identification of vaginal secretion or menstrual blood as cellular origin of detected DNA by analyzing a specific CpG site can offer a meaningful assessment of potential vaginal penetration. This CpG site V2 [1] was also investigated in other studies, all studies comprising women between 18 and 94 years [4, 11, 15], however, no information was available in these studies whether or how many female DNA donors had already reached the menopause.

It has already been shown that the epigenome, especially DNA methylation, is fundamentally regulated or altered by hormones [16]. In addition, a study has shown that DNA methylation changes depend on a woman’s hormone status (before, during and after sexual maturity) [17]. In addition, differences in methylation patterns can be observed with hormone substitutions, e.g. during menopause [18].

Since there are several cases of sexual assault involving victims beyond an age in which menopause could be assumed, the aim of this study was to include additional markers in the existing BFI workflow to get an extended version, (eBFI workflow) that reliably identifies vaginal secretions regardless of a woman’s age. Not only women who have already reached menopause, but also girls before reaching sexual maturity were explicitly included. Since we observed during this study that the existing blood marker B7 was sometimes unable to provide clear results, an additional blood marker was added to the extended version of the workflow (eBFI).

## Materials and methods

### Samples

The study comprised 381 adult samples from different origins: nasal samples (including nasal mucosa and nasal blood samples, 65), oral mucosa / saliva samples (64), blood samples (73), vaginal fluid samples (53), menstruation blood samples (42) and semen samples (45) and additionally 39 vaginal fluid samples of women who reached menopause (collected between 2023 and 2024 in the Institute of Legal Medicine, Essen and by medical specialists in gynecology). All study participants were between the ages of 18 and 69, consisting of 92 women and 45 men, who voluntarily provided their samples. No exact specifications were given for sampling, as this procedure best represents casework samples. The samples described above included ten blood samples and ten vaginal secretion samples which were used exclusively for the validation of the new CpG markers B6 and V1. The validation was performed by two scientists independently without knowledge of samples’ origin.

Additionally, 30 samples from 21 young girls under 10 years and 42 samples from 20 young girls between 11 and 14 years were included. These samples were taken by a gynecologist after sexual assaults against underage girls between 2007 and 2024 as part of preservation of evidence. After conclusion of the respective criminal proceedings, the real case work samples were released for research by the responsible public prosecutor’s offices, resulting in a total of 453 analyzed samples.

No information was available about last sexual intercourse, diseases or operations like hysterectomy; two females (at the age of 66 years and 56 years) reported taking hormones.

### Compliance with ethical standards

All samples were obtained after informed consent or after release of the public prosecutor’s offices and with approval of the Medical Ethics Committee at the University of Duisburg-Essen in accordance with the Declaration of Helsinki and national laws (ethic vote number: 21-9843-BO).

### Selection of potential additional CpG markers

V1 was selected as a second vaginal secretion-specific marker and B6 was taken as an additional blood specific marker. Based on existing literature in context of body fluid identification analysis [4, 15, 19, 20], both markers were already tested in experiments during our prior study [1], but have not been selected for the final BFI workflow. This workflow included marker NB21 as nasal blood specific marker; B7 as blood specific markers; MB4 as menstrual blood specific marker; SA4 as saliva specific marker; V2 as vaginal secretion specific markers; N27SE as nasal secretion und semen specific marker, the associated genes or genome positions and further information can be found in the previous study and in Table S1 [1].

V1 is located in a CpG Island at chromosome 7 (chr7:27.251.719–27.251.720) and thus in an epigenetically modified accessible region of the transcription factor EVX1 [21] which is involved in the regulation of transcription of HOXA cluster. HOXA10 and HOXA11 are important factors in fertility in women, since both are critical for implantation of a blastocyst [22–24].

B6 is located within the gene S1PR4 (chr19:3.178.957–3.178.958), which codes for a G-protein-coupled receptor protein, more precisely for the sphingosine-1-phosphate receptor 4. S1PR4, along with other variants of sphingosine-1-phosphate receptors, regulates lymphatic migration [25, 26].

Information about all markers used in the eBFI workflow is summarized in Table S1.

### Primer and assay design, DNA extraction, quantification, bisulfite conversion, amplification and sequencing

The PCR and sequencing primers were successfully designed using PyroMark assays Design 2.0 software (Qiagen, Hilden, Germany) using parameters as suggested in manufactures’ instructions. Melting temperatures of PCR primers varied between 55.1 °C and 62.6 °C, melting temperature of sequencing primers was between 37.1 °C and 51.8 °C. Details on primer sequences will be provided by the authors on request. The assay design for pyrosequencing was realized with PyroMark Q48 Autoprep software (Qiagen) to be as short as possible dispensations between 13 nucleotides and 22 nucleotides. Each sequencing assay contained a bisulfite conversion control site. DNA extraction was performed using the DNA IQ Casework Pro Kit and Casework Extraction Kit in the Maxwell^®^ RSC 48 instrument (Promega, Walldorf, Germany). Subsequently, DNA was quantified using the PowerQuant™ System (Promega) and converted using the MethylEdge Conversion System Kit (Promega). Pyrosequencing was done using PyroMark^®^ PCR (Qiagen) and the PyroMark Q48 Autoprep instrument (Qiagen) plus using PyroMark^®^ Q48 CpG Reagent Kit (Qiagen). In addition, the EpiTect PCR Control DNA Set (Qiagen) comprising both bisulfite converted methylated and unmethylated DNA and unconverted unmethylated DNA was used for monitoring of complete bisulfite conversion. Methylation calculation was done using the software PyroMark Q48 Autoprep (Qiagen). The data analysis was carried out with Excel (Microsoft) and displayed using boxplots. All methods were performed as described in our previous study [10].

## Results and discussion

### Reliability of data

All samples used in this study were processed in exactly the same way. Therefore, influence of extraction methodology could be excluded. Only samples with a minimum concentration of 0.75 ng/µl were considered, as only they met the defined input quantity of DNA for bisulfite conversion. All bisulfite conversion controls during pyrosequencing were negative as desired, demonstrating successful conversion for all samples. Basically, all samples were analyzed as duplicates and the deviation rate was a maximum of 5%.

### Establishment and validation of additional vaginal marker and blood marker

**Fig. 1:**
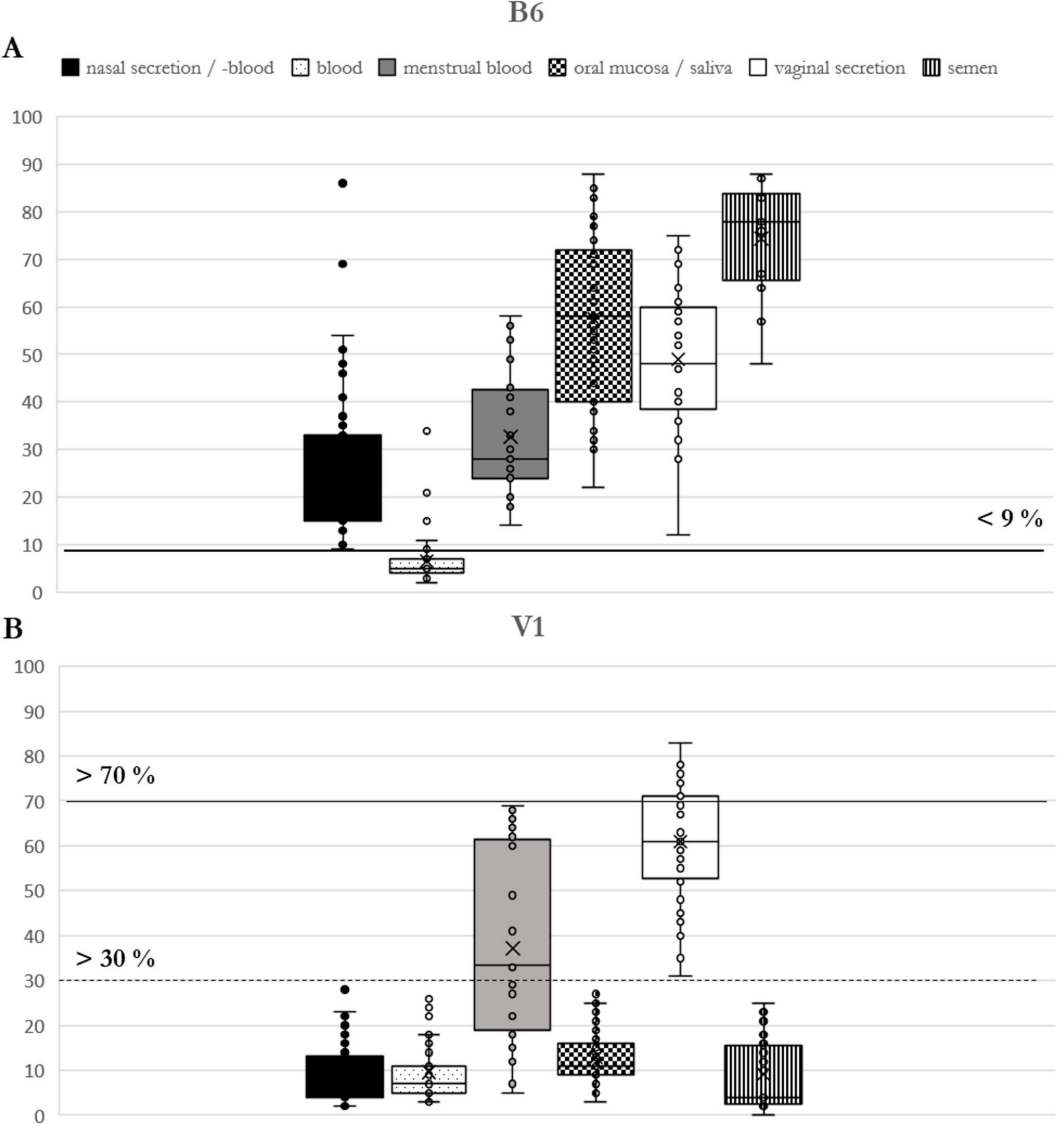
(**A**) Blood marker B6 box plot. (**B**) Vaginal secretion marker V1 box plot. The box plots show the different methylation levels of the various body fluids (establishment cohorts only) for the respective CpG marker. The black lines define cut-off values (without pre-tests), the dashed line (in B) indicates the threshold value under the assumption of a negative blood pre-test result. These critical values determine the methylation range in which the target secretion could be clearly identified. Below (V1) or above (B6) these thresholds, the target secretion may be present, but a different origin or mixtures are also possible

As for marker B6, DNA methylation percentage of peripheral blood (establishment cohort: 46 samples from donors who were at least 18 years old) ranged between 2% and 34% with a mean value of 7% and a standard deviation of 5% in the establishment cohort. All other secretions reached values between 9% (nasal blood and mucosa samples) and 88% (saliva and sperm secretion). Consequently, a threshold for identification of blood could be established at < 9% (Fig. 1A).

In the subsequent validation study, ten out of ten blood samples were identified as true positive, false positive results did occur for two nasal mucosa samples. To avoid misinterpretation, the second blood marker B7 should also show a positive result for blood, and the nasal secretion marker should additionally refute the presence of the target secretion (Table S2). If the result is inconclusive, the conclusion should always be that blood could not be reliably detected.

Regarding marker V1, DNA methylation percentage of vaginal secretion (initial test cohort) varied between 31% and 83% with a mean value of 61% and a standard derivation of 13%. All other secretions reached methylation values between 2% (nasal samples) and 69% (menstrual blood). Therefore, a threshold for identification of vaginal secretion in samples with negative blood pretest result could be established at > 30% (Fig. 1B). In case of positive blood pretests, DNA could have its origin either in vaginal secretion or in menstrual blood. But this fact is no problem in context of sexual assaults, since both secretions originate in the vaginal tract and can prove sexual contact.

In the validation study, it was possible to identify ten out of ten vaginal secretion samples as true positive. There were no false positive results regarding all other secretions during validation except menstrual blood samples, here ~ 27% showed false positive results.

**Fig. 2:**
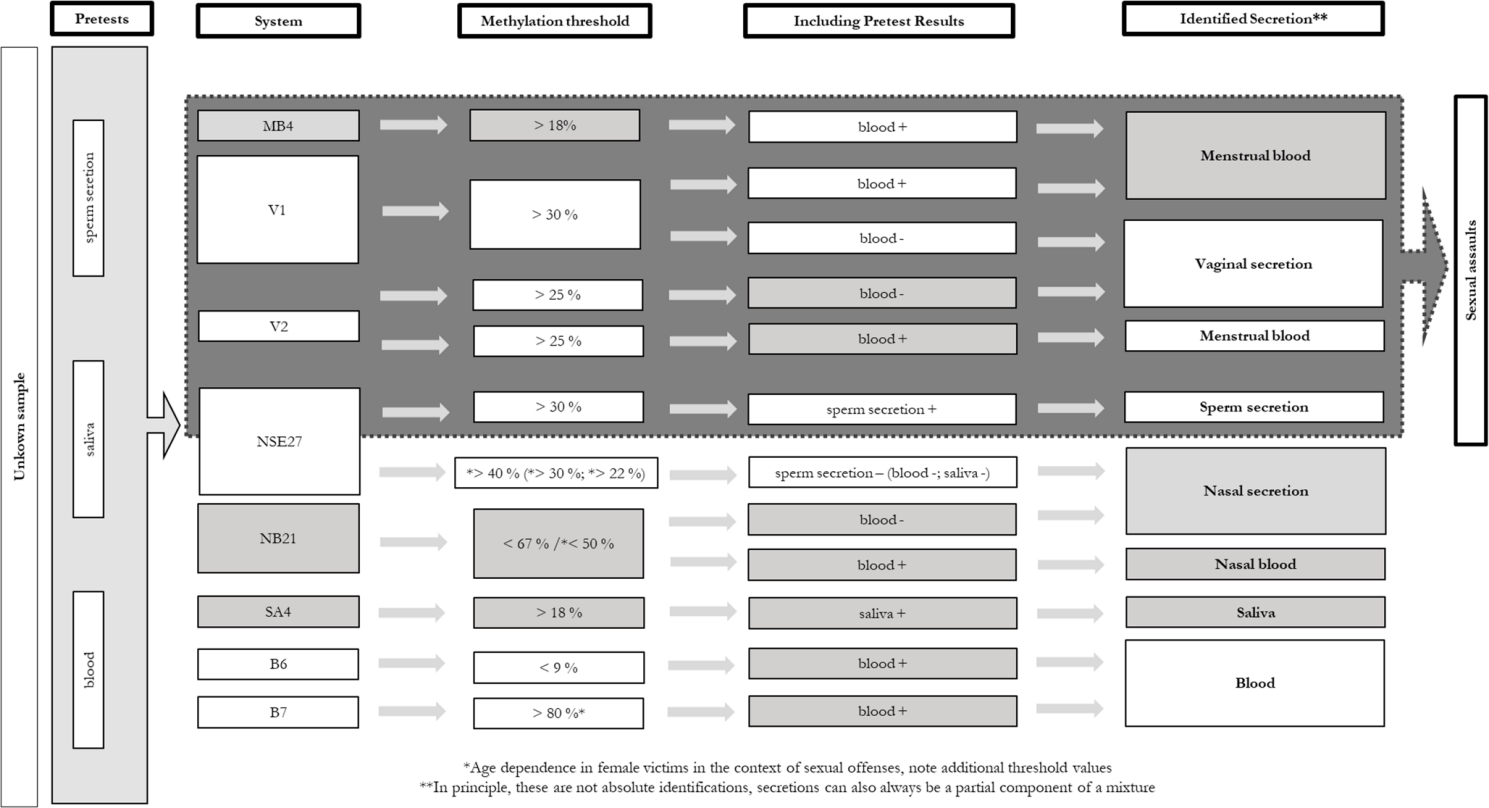
The extended eBFI workflow. Extension of the published BFI workflow [1] for identification of body fluids using pre-tests/rapid tests and methylation analysis; the method enables the direct identification of seven different body fluids and mixtures thereof. (*NB21* nasal blood specific marker, *B7* and *B6* blood specific markers, *MB4* menstrual blood specific marker, *SA4* saliva specific marker, *V1* and *V2* vaginal secretion specific markers, *N27SE* nasal secretion und semen specific marker)

Regarding the two new markers, we applied the same rule for evaluation of results as for markers from BFI workflow: below (V1) or above (B6) the described thresholds, the target secretion may be present, but a different origin is also possible. Therefore, both CpG markers were added in the eBFI workflow so that it now comprises a total of eight markers (NB21: nasal blood-specific marker; B7 and B6: blood-specific markers; MB4: menstrual blood-specific marker; SA4: saliva-specific marker; V1 and V2: vaginal secretion-specific markers; N27SE: nasal secretion- and sperm-specific marker, Fig. 2).

### The problem of hormone status

In one of our routine cases, the female victim of suspected rape was over 80 years old. Here, the hypothesis arose that the methylation percentage of marker V2 in vaginal secretion could differ significantly from established values due to differences in hormone status. A very small sample cohort (*n* = 4) consisting of vaginal swab samples exclusively from women beyond menopause showed significantly lower methylation results in assay V2 compared to the samples of the establishment and validation cohort comprising women predominantly of childbearing age. Using the established cut-off value of > 50% [1], none of the four samples would have been identified as vaginal secretion. It was assumed that a functional change could also cause a difference in the methylation percentage of specifically selected CpGs in vaginal secretion thus significantly reducing the power of our analysis. Moreover, this sample’s result also raises the question about the methylation percentage in girls too young for sexual maturity.

Therefore, samples were divided into four cohorts: DNA extracted from vaginal secretions of girls under 10 years, girls between 11 and 14 years, women between 18 and 60 years and women older than 60 years. Individuals in the last cohort voluntarily confirmed to have reached menopause.

 In general, women reach menopause at an age between 45 and 60 years, with an average age of 51 years [27]. So, several women of our first study [1] may have been in menopause, since no such information was collected in this study.

**Fig. 3:**
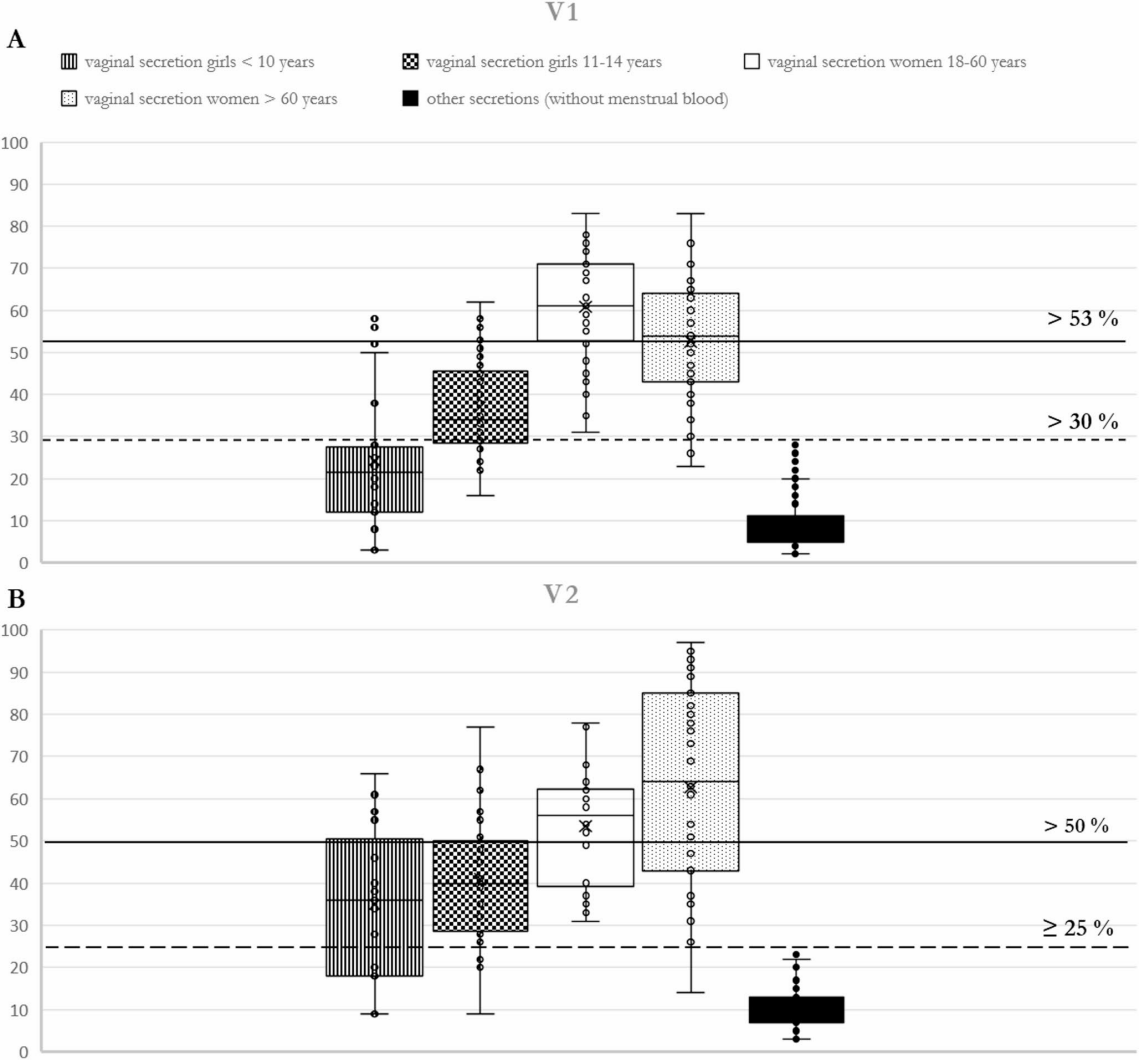
(**A**) V1 box plot. (**B**) V2 box plot. The box plots show different methylation levels of the four female cohorts for vaginal secretion in vaginal secretion specific markers V1 and V2 in contrast to results of all other secretions (without menstrual blood). The black lines indicate thresholds for absolute identification of a secretion out of the vaginal tract (vaginal secretion or menstrual blood). The dashed lines indicate threshold for identification of vaginal secretion as a component of a mixture. The third cohort, vaginal secretion women 18 - 60 years, is representing the initially analyzed group for establishment of the markers [1]

The results of the four cohorts were plotted together with the results of marker establishment in box plot diagrams and compared with the respective reference range and with the respective threshold values of the corresponding target secretion (Fig. 3 A & B).

The newly added vaginal secretion marker V1 also showed a shift in methylation percentage depending on the age of the test subjects (Fig. 3 A). Initially it increases with age and then decreases again (Fig. 3 A). The threshold value for identifying vaginal secretions or menstrual blood with this assay is > 30%. Taking this threshold value into account, only around 53% of juvenile vaginal secretion samples (girls < ten years and girls between eleven and 14 years) and 97% of adult female samples could be identified as vaginal secretions (Table 1).

**Table 1:** Methylation percentages mean values and standard deviation of four cohorts of vaginal secretion (women and girls of different ages). The proportion of identifiable samples with the respective marker based on the respective threshold is also given

		< 10 years	11–14 years	18–60 years		> 60 years
V1	mean value and standard deviation	24.1 ± 16.4	37.5 ± 10	60.9 ± 13.1		52.6 ± 13.9
	identified samples ( threshold > 30%)	53%		97%		
V2	mean value and standard deviation	34.9 ± 17.5	41 ± 15.4	53.5 ± 13.4		62.7 ± 23.1
	identified samples ( threshold > 25%)	84%		98%		
V1 & V2	identified samples	91%		99%		

As assumed, marker V2 showed a wider range of methylation percentage if including young girls and women in menopause (Fig. 3 B). It increases with increasing age (Fig. 3 B). The threshold value for identifying vaginal secretion or menstrual blood with this assay is > 25%. Taking this threshold value into account, around 84% of the juvenile vaginal secretion samples (girls < ten years and girls between eleven and 14 years) can be identified, compared to 98% of the samples from adult women. (Table 1) thus supporting our theory about a dependence on hormone status for both vaginal markers.

As a reversible epigenetic mechanism, DNA methylation controls both differentiation of cell types and their functionality. Several changes in a women’s body depend on the hormone status, such as fertility. Although V1 and V2 are not gene associated, they are located in epigenetically modified accessible regions for HOX clusters A and C. HOX genes are not only significantly involved in differentiation during embryonic development (morphogenesis) [28], but HOXA10, HOXA11, HOXC10 and HOXC11, among others, are also crucial for female fertility. The expression of these genes could be detected in the endometrium during the entire menstrual cycle [29] and they are essential for successful implantation [30]. Therefore, it is not surprising that methylation patterns of V1 and V2 show differences depending on a woman’s age or hormone status.

The combination of V1 and V2 increases the proportion of samples successfully identified as vaginal secretions or menstrual blood, so that 91% of samples from underage girls and 99% of samples from adult females could be correctly identified by analyzing both markers (Table 1).

### Methylation analysis of vaginal secretion from girls and older women in the eBFI workflow

The analysis of the four different cohorts of vaginal secretions with established BFI workflow [1] resulted in adjustment of some threshold values for non-vaginal markers. The results are presented in box plots showing different methylation levels of the four female cohorts for vaginal secretion in the BFI workflow markers in contrast to summarized results of all other secretions (except for the respective target secretion). The area shaded in grey represents the methylation percentage of target secretions. The black and the dashed lines (additionally pre-test result(s) necessary) indicate different threshold values for identification of the respective target secretion (absolute or as part of a mixture; Figure S1).

 Based on the new validation results, the threshold value for identification of nasal blood or nasal secretions with marker NB21 must be reduced to < 50% (Fig. 2 eBFI workflow and Figure S1 A).

Due to validation with the new cohorts, the marker B7 is no longer usable in some cases (Fig. 2 eBFI workflow and Figure S1 B). Therefore, a new blood marker, B6, was established and validated (see above and Fig. 2 eBFI workflow and Figure S1 C).

The cut-off value for identifying menstrual blood with marker MB 4 is not affected by the results. Only exception is an outlier in the first cohort with 23%. As already described for marker V1, it is not necessary to differentiate between vaginal secretions and menstrual blood in the context of sexual offenses, as both secretions only occur in the vaginal tract. In addition, the result of a blood pretest easily enables a distinction (Fig. 2 eBFI workflow and Figure S1 D).

The threshold value for identifying saliva with marker SA4 was only compromised by a single outlier from the cohort of women older than 60 years (Fig. 2 eBFI workflow and Figure S1 E). However, if a positive result in the methylation analysis is combined with a positive result in the pre-test, a false positive identification of saliva can be ruled out.

In contrast, N27SE marker is only suitable for detection of nasal secretions in connection with a sexual offense if the age of an involved woman is between 18 and 60 years. Under these conditions, the thresholds remain unchanged, otherwise identification of nasal secretion with this marker is no longer possible. With regard to the identification of sperm secretions, the threshold remains unchanged in either scenario (Fig. 2 eBFI workflow, Figure S1 F).

Raw methylation data can be provided by the authors on request.

## Conclusion

In this study, the existing BFI workflow for identification of seven different body fluids was further improved by adding two additional markers. The complementary use of two vaginal markers and two blood markers increases not only the power of our eBFI workflow, but enables the identification of vaginal secretions and blood regardless of hormone status or age of the victim.

However, the inclusion of so far not tested kinds of samples also resulted in an adjustment of established threshold values. Here, the analysis of other different secretions or cell types is under way to further improve the informative value of the eBFI workflow. In addition, no tests were carried out with vaginal secretions from girls in puberty (15–17 years). Results of such samples could also lead to further changes with regard to the threshold values.

Moreover, there is a critical limitation in the sample material itself. Real casework samples are not subject to standardized sampling routines or defined storage conditions. In 2024, Ghemrawi et al. showed for saliva samples that the method of sampling and the storage of material can have an influence on the methylation value [31]. But the non-standardized sampling and storage conditions in our study should be more representative of casework samples and counteract this problem at least partially. 

## Supplementary Information

Below is the link to the electronic supplementary material.

Supplementary File 1 (DOCX 162 KB)

Supplementary File 2 (DOCX 14.3 KB)

Supplementary File 3 (DOCX 16.0 KB)
