# Supplementary material for: Pitfalls in body fluid identification – age independent DNA methylation markers for vaginal secretions and menstrual blood in sexual assaults
Source: Int J Legal Med. 2026 Feb 19;140(3):1339–47. doi: 10.1007/s00414-026-03717-0 (PMC13161311; doi:10.1007/s00414-026-03717-0)
Supplement: Supplementary file 1 — (DOCX 162 KB) [file 414_2026_3717_MOESM1_ESM.docx]

**Figure S1:**

The box plots presented the different methylation levels of the four female cohorts for vaginal secretion in the BFI workflow markers [9] in contrast to summarized results of all other secretions (except for the respective target secretion). The area shaded in grey represents the methylation percentage of target secretions. The black and the dashed lines (additionally pre-test result(s) necessary) indicate different threshold values for identification of the respective target secretion, absolute or as part of a mixture. (NB21: nasal blood specific marker; B7 and B6: blood specific markers; MB4: menstrual blood specific marker; SA4: saliva specific marker; N27SE: nasal secretion und semen specific marker).

**
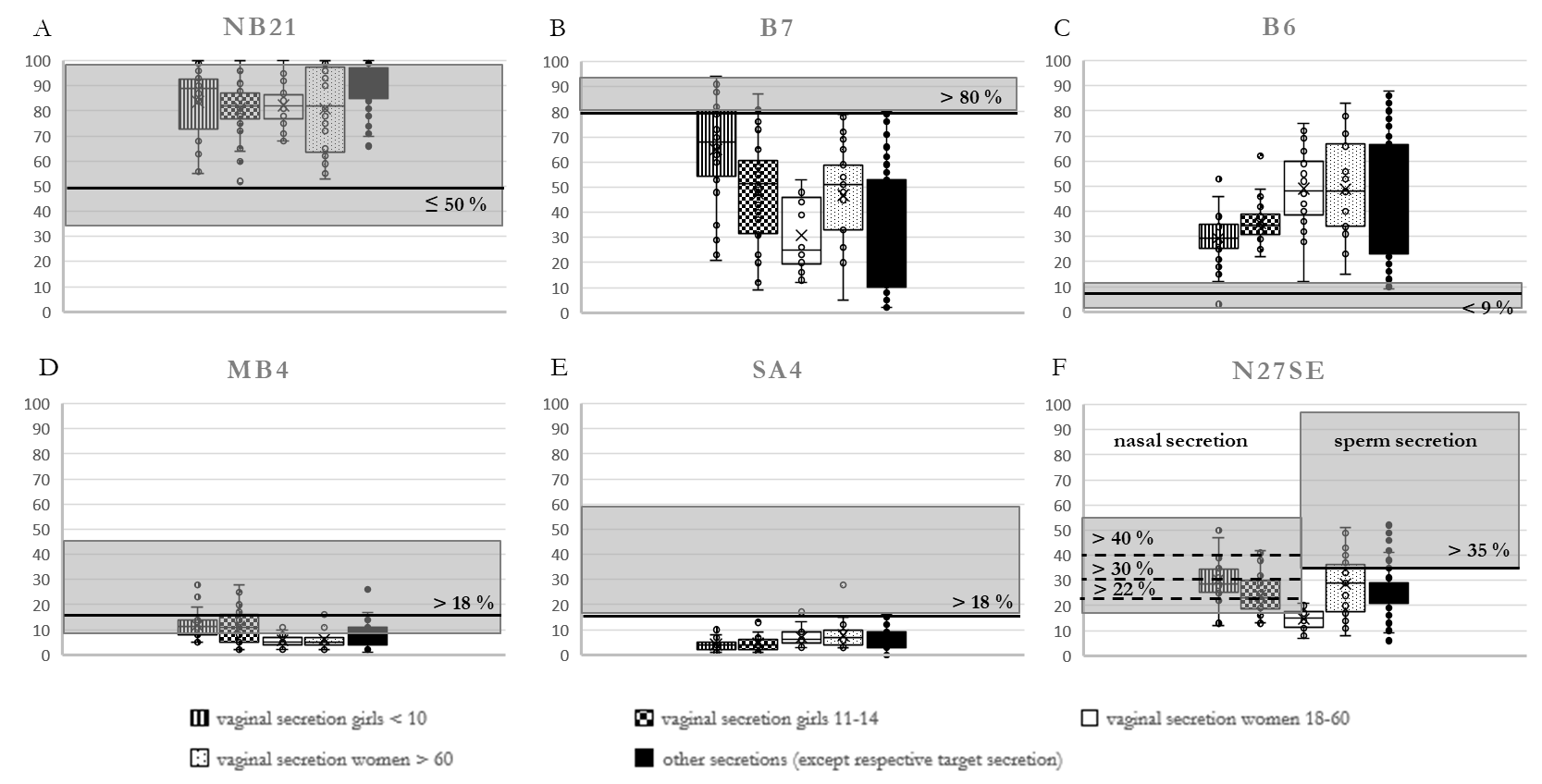
**
