# Supplementary material for: Pitfalls in body fluid identification – age independent DNA methylation markers for vaginal secretions and menstrual blood in sexual assaults
Source: Int J Legal Med. 2026 Feb 19;140(3):1339–47. doi: 10.1007/s00414-026-03717-0 (PMC13161311; doi:10.1007/s00414-026-03717-0)
Supplement: Supplementary file 2 — (DOCX 14.3 KB) [file 414_2026_3717_MOESM2_ESM.docx]

**Table S1:** Information on the CpG ID, the associated gene, the gene function, the exact genome locus (GRCh38) and the study in which it was first published in the context of body fluid identification for each eBFI workflow marker.

| **marker** | **CpG ID** | **gene** | **gene function** | **locus (GRCh38)** | **published in context of body fluid identification:** |
| --- | --- | --- | --- | --- | --- |
| NB_21 | cg16518142 | CDH26 | cell adhesion molecule, extracellular matrix | chr20:59.958.658-59.958.659 | Konrad et al. 2023 |
| N_27_SE | cg20864568 | MAP3K14 | signal transduction/ cell proliferation | chr17:45.314.233-45.314.234 | Konrad et al. 2023 |
| SA_4 | cg21597595 | unclassified | - | chr2:5.366.095-5.366.096 | Forat et al. 2016 |
| B_6 | cg17518965 | S1PR4 | G protein-coupled receptor protein | chr19:3.178.957-3.178.958 | Vidaki et al. 2016 |
| B_7 | cg13763232 | SLC6A6 | Sodium- and chloride-dependent taurine transporter | chr3:14.401.928-14.401.929 | Vidaki et al. 2016 |
| V_1 | cg09765089 | unclassified | - | chr7:27.251.719-27.251.720 | Lee et al. 2015; Lee et al 2016; Lin et al. 2016 |
| V_2 | cg26079753 | unclassified | - | chr12:53.961.744-53.961.745 | Lee et al. 2015; Lee et al 2016 |
| MB_4 | cg04255276 | LTBP3 | Latent-transforming growth factor beta-binding protein 3 | chr11:65.546.550-65.546.551 | Lee et al 2016 |
