# Supplementary material for: Pitfalls in body fluid identification – age independent DNA methylation markers for vaginal secretions and menstrual blood in sexual assaults
Source: Int J Legal Med. 2026 Feb 19;140(3):1339–47. doi: 10.1007/s00414-026-03717-0 (PMC13161311; doi:10.1007/s00414-026-03717-0)
Supplement: Supplementary file 3 — (DOCX 16.0 KB) [file 414_2026_3717_MOESM3_ESM.docx]

**Table S2:** Results of the validation of the two additional markers for the eBFI workflow V1 and B6. (n: nasal blood und mucosa; b: blood; mb: menstrual blood; sa: saliva; v: vaginal secretion; se: sperm secretion)

| **V1** | **n** | **b** | **mb** | **sa** | **v** | **se** |
| --- | --- | --- | --- | --- | --- | --- |
| **n** | **22** | **22** | **15** | **21** | **10** | **9** |
| **true positive** | - | - | - | - | **10** | - |
| **in %** | - | - | - | - | **100** |  |
| **false positive** | 0 | 0 | 4 | 0 | **-** | 0 |
| **in %** | 0 | 0 | 26,67 | 0 | **-** | 0 |
| **true negative** | 22 | 22 | 11 | 21 | **-** | 9 |
| **in %** | 100 | 100 | 73,34 | 100 | **-** | 100 |
| **false negative** | - | - | - | - | **0** | - |
| **in %** | - | - | - | - | **0** | - |
| **B6** | **n** | **b** | **mb** | **sa** | **v** | **se** |
| **n** | **10** | **10** | **7** | **10** | **5** | **10** |
| **true positive** | - | **10** | - | - | - | - |
| **in %** | - | **100** | - | - | - | - |
| **false positive** | 2 | **-** | 0 | 0 | 0 | 0 |
| **in %** | 20 | **-** | 0 | 0 | 0 | 0 |
| **true negative** | 8 | **-** | 7 | 10 | 5 | 10 |
| **in %** | 80 | **-** | 100 | 100 | 100 | 100 |
| **false negative** | - | **0** | - | - | - | - |
| **in %** | - | **0** | - | - | - | - |
